# Cultured Alveolar Epithelial Cells From Septic Rats Mimic *In Vivo* Septic Lung

**DOI:** 10.1371/journal.pone.0011322

**Published:** 2010-06-25

**Authors:** Taylor S. Cohen, Gladys Gray Lawrence, Susan S. Margulies

**Affiliations:** Department of Bioengineering, University of Pennsylvania, Philadelphia, Pennsylvania, United States of America; University of Giessen Lung Center, Germany

## Abstract

Sepsis results in the formation of pulmonary edema by increasing in epithelial permeability. Therefore we hypothesized that alveolar epithelial cells isolated from septic animals develop tight junctions with different protein composition and reduced barrier function relative to alveolar epithelial cells from healthy animals. Male rats (200–300g) were sacrificed 24 hours after cecal ligation and double puncture (2CLP) or sham surgery. Alveolar epithelial cells were isolated and plated on fibronectin-coated flexible membranes or permeable, non-flexible transwell substrates. After a 5 day culture period, cells were either lysed for western analysis of tight junction protein expressin (claudin 3, 4, 5, 7, 8, and 18, occludin, ZO-1, and JAM-A) and MAPk (JNK, ERK, an p38) signaling activation, or barrier function was examined by measuring transepithelial resistance (TER) or the flux of two molecular tracers (5 and 20 Å). Inhibitors of JNK (SP600125, 20 µM) and ERK (U0126, 10 µM) were used to determine the role of these pathways in sepsis induced epithelial barrier dysfunction. Expression of claudin 4, claudin 18, and occludin was significantly lower, and activation of JNK and ERK signaling pathways was significantly increased in 2CLP monolayers, relative to sham monolayers. Transepithelial resistance of the 2CLP monolayers was reduced significantly compared to sham (769 and 1234 ohm-cm^2^, respectively), however no significant difference in the flux of either tracer was observed. Inhibition of ERK, not JNK, significantly increased TER and expression of claudin 4 in 2CLP monolayers, and prevented significant differences in claudin 18 expression between 2CLP and sham monolayers. We conclude that alveolar epithelial cells isolated from septic animals form confluent monolayers with impaired barrier function compared to healthy monolayers, and inhibition of ERK signaling partially reverses differences between these monolayers. This model provides a unique preparation for probing the mechanisms by which sepsis alters alveolar epithelium.

## Introduction

Acute lung injury (ALI) and acute respiratory distress syndrome (ARDS) affect 1.5–75 cases per 100,000 people annually, with mortality rates of 25–40% [1], [2], [3], [4]. ALI can be induced by a broad spectrum of insults, including large tidal volume ventilation, pneumonia, ischemia, smoke inhalation, pulmonary hemorrhage, and sepsis [5], [6]. Characterized by an acute onset, severe hypoxemia, left atrial hypertension, and pulmonary edema, ALI can lead to multiple organ failure and death (See Wheeler *et. al.* for a detailed overview of ALI, its symptoms, and current treatment strategies) [5].

Sepsis, of either pulmonary or non-pulmonary origin, is the post common clinical precursor to ALI, accounting for 25–40% of ALI cases [7], [8]. One hallmark of both sepsis and ALI is a breakdown of the alveolar epithelial barrier (due to alveolar epithelial type I cell loss), accompanied by a loss of barrier function and the development of alveolar edema [5], [9]. Techniques involving *in vivo* confocal microscopy to view subpleural alveoli or labeling of fixed lung slices are currently used to study these cells in the intact organ [10], [11], [12], [13]. Alternatively, homogenates of the lung have been used to probe for activation of signaling pathways in the lungs of septic animals [14]. Studies of this nature are limited by the inability to differentiate responses and mechanisms that may be specific to cell type (e.g. endothelial, epithelial type I, epithelial type II, airway epithelial, macrophages, etc.) [15].

Cell culture models of alveolar epithelia, either primary culture or immortalized cell line, have advantages over whole organ models including controllable conditions, repeatable injuries and treatments, lower costs, and high study throughput. In the study of ALI, culture models have been used to identify mechanisms, including signaling activation, increased cell mortality, and protein alterations, by which epithelial cells respond to various environmental mediators found in the injured lung such as hypoxia, mechanical stretch, inflammatory mediators, or bacterial toxins [16], [17], [18], [19], [20], [21], [22]. However cell culture models cannot reproduce the injurious stimuli experienced *in vivo*, which is a combination of the initial stimulus (ventilation, hypoxia, pneumonia) and the coordinated inflammatory response of numerous cell types, including epithelial cells, macrophages, and neutrophils.

In this communication we test the hypothesis that alveolar epithelial cells isolated from septic rats will retain a septic phenotype in culture, including increased mitogen activated protein kinase (MAPk) signaling activity, altered tight junction structure, and decreased barrier function [9], [13], [14]. We present a novel culture model of sepsis-induced ALI where the septic insult is applied *in vivo*, prior to cell isolation. We show that primary alveolar epithelial cells (AEC) isolated from septic or sham control rats form confluent monolayers with intact tight junctions and express type I phenotypic markers by day 5 in culture. However, monolayers formed by epithelial cells isolated from septic animals develop “leakier” tight junctions, have altered expression of tight junction proteins, and exhibit elevated activation of MAPk signaling pathways compared to monolayers composed of cells isolated from healthy animals, all symptoms of ALI. We demonstrate that inhibition of MAPk signaling leads to partial recovery of barrier function in septic monolayers, potentially through alterations in tight junction protein expression. These data show that this cell culture model mimics an *in vivo* septic epithelium, and responses to interventions shown to reduce septic injury *in vivo*. Finally, we identified a mechanism through which sepsis produces epithelial barrier dysfunction by demonstrating that altered expression of key tight junction proteins in our septic epithelium are reversed with ERK MAPk inhibition.

## Methods

### Ethics Statement

All animal use was done in accordance with, and with the approval of, the IACUC in the Office of Regulatory Affairs of the University of Pennsylvania.

### Cecal Ligation and Double Puncture (2CLP)

Under sterile conditions and isoflurane anesthesia, male Sprague-Dawley rats (Charles River, Boston, MA) weighing 240–260 grams were underwent 2CLP as previously described [23]. Briefly, the abdomen was opened via midline abdominal incision, the cecum exposed and ligated distal to the ileocecal valve, so as not to obstruct the intestine, and two punctures were made with an 18 gauge needle, which allowed a small amount of fecal matter to be extruded. The cecum was then placed back in the animal, the abdomen was closed, and the animal was returned to a clean cage with full access to food and water. Animals were treated just following surgery, and then again 12 hours later, with a 0.4 ml/kg subcutaneous dose of buprenorphine for analgesia. Parallel sham animals were subjected to all aspects of the procedure except for the cecal ligation and puncture. Mortality rate 24 hours following the 2CLP procedure was approximately 10%. All methods were approved by IACUC of the University of Pennsylvania.

### Alveolar Epithelial Type II Cell Isolation

Twenty-four hours following the 2CLP or sham procedures, animals were observed for signs of distress (lethargy, porphyrin staining of the eyes and nose, tissue dehydration and inflammation), anesthetized (sodium pentobarbital, 55 mg/kg ip), the trachea cannulated, and the lungs mechanically ventilated. Blood samples were obtained via the descending aorta with a heparin coated 21 gauge needle for analysis of hematocrit, platelet, neutrophil, lymphocyte, white blood cells, and cytokine expression. Cytokine expression was analyzed with a fluorescent glass slide array (Rat Cytokine Array G, RayBiotech, Norcross, GA). Slides were imaged and cytokine expression quantified on a fluorescent scanner (Axon GenePix, Molecular Devices, Sunnyvale, CA).

An abdominal aortotomy was performed to exsanguinate the rat, and excess blood removed via pulmonary arterial perfusion. The lungs were excised, bronchial lavage samples were collected for analysis of cellular content via a tracheal instillation and withdrawal (repeated 3 times) of 7 mls of physiologic saline, and type II cells isolated using an elastase digestion technique [24]. Briefly, the airways were infused with elastase and incubated at 37 deg C for 1 hour, after which they were diced, and the tissue slurry was filtered through consecutively smaller filters. IgG panning was used to remove all but the alveolar epithelial type II cells (purity >90%). Cells were re-suspended in a solution of minimum essential medium (MEM), 10% fetal bovine serum (FBS), 0.4 µl/ml Gentamicin, and 1µl/ml Amphotericin B (Life Technologies, Rockville, MD) and seeded at 1×10^6^ cells/cm^2^ onto fibronectin coated (10 µg/cm^2^) flexible silastic membranes (Specialty Manufacturing, Saginaw, MI) in custom-designed wells, or transwell permeable supports (PCF membrane, pore size 0.4 µm^2^, Corning Inc. Corning, NY). Cells were also seeded at 1×10^5^ cells/cm^2^ on fibronectin coated glass slides for cell spreading experiments. The media was replaced daily, and cells were used on either the second day for spreading measurements, or the fifth day for all other experiments.

### Phenotypic and Immunohistological Characterization

After 2 and 5 days in culture, sham and 2CLP cells were fixed with methanol (4°C), washed with phosphate buffered saline (PBS), and incubated overnight (4°C) with primary antibodies for phenotypic markers of alveolar epithelial type II and type I cells (N≥3 rats/marker). For type II: Tuloidin Blue lamellar body stain (Sigma Chemical, St. Louis, MO). For type I: RT1-40 (gift from Dr. L. Dobbs, UCSF), plasminogen activator inhibitor-1 (PAI-1, gift from C. Foster, CHOP). Fluorescently conjugated secondary antibodies were used to image the staining patterns. For all imunohistochemistry, images were obtained using confocal microscopy with 60× magnification. To further confirm this phenotypic transformation we embedded monolayers from 2CLP and sham rats maintained in culture for 2 and 5 days in Epon A12 (Electron Microscopy Supply, Port Washington). Blocks were sectioned (60–85 nm) and imaged using electron microscopy (2500×–150000×, 15–20 regions/well, 1 well each condition).

### Cell Spreading Analysis

Cells plated on glass slides were fixed following 48 hours in culture with 1.5% paraformaldehyde for 15 minutes, washed with PBS, and stained for actin by overnight incubation (4 degrees C) with FITC labeled phalloidin (Sigma, Saint Louis, MO). Slides were imaged (Nikon TE-300, 60× objective), cell borders (labeled by actin) were traced and cell area calculated using NIH Image J. Pixel number was translated to area (µm^2^) via a calibrated glass slide where, at this magnification and resolution, 93 pixels was equivalent to 10 µm. Area measurements for sham (N = 25) and 2CLP (N = 18) cells were compared using a Student's t-test (p<0.05).

### Transepithelial Resistance (TER) Measurements

TER measurements (N≥10 wells per group) correlate with ion motion across the epithelial membrane, with an inverse relationship between TER and ionic flux [25]. Cell monolayers plated on transwell filters were serum deprived for 2 hours in Dulbecco's Modified Eagle Medium (DMEM) with HEPES, after which the basal and apical media was changed and TER was measured (0, 60, 90, and 120 minutes following this switch) using a Millicell-ERS system (Millipore, Bedford, MA). TER was compared between 2CLP and sham groups using a Student's t-test (p<0.05).

To determine the TER measured was due to transcellular or paracellular pathways, 2CLP and sham monolayers (N≥8 wells per group) were treated with specific inhibitors of ENaC and Na^+^/K^+^-ATPase, two transcellular pathways for ion motion across the epithelium (N≥8 per treatment). Following initial TER measurements in DMEM, the apical fluid was removed and replaced with DMEM containing amiloride (1 mM) or the basal fluid removed and replaced with DMEM containing ouabain (1 mM) (Sigma, Saint Louis, MO). In control wells both the apical and basal fluids were removed and replaced with DMEM as a loading control. Following a 10 minute incubation period, TER values were measured and normalized to the pre-treated TER values of the individual well. An ANOVA was used to determine significance with post-hoc Dunnett's tests to compare the effect of treatments to the control in each group, 2CLP or sham (p<0.05).

### Monolayer Permeability to Carboxyfluorescein and BODIPY-Ouabain

Paracellular permeability (P) was assessed in 2CLP and sham monolayers plated on the permeable membrane by monitoring flux of the fluorescent tracer carboxyfluorescein (approximate radius 5 Å, Sigma, Saint Louis, MO) or on the non-permeable silastic membrane by monitoring the presence of BODIPY-Ouabain (approximate radius 15 Å, Molecular Probes, Eugene, OR) at the basal surface of the cell as described previously [17], [26]. To determine carboxyfluorescein permeability (N≥12 wells per group: 3 isolations, 4 wells/isolation) cells were mounted into a Ussing system with the basal chamber filled with 500 µl Ringer's solution, and the apical chamber filled with Ringer's spiked with 10% wt/volume carboxyfluorescein. Basal samples (200 µl) were drawn at 0 and 120 minutes after mounting and replaced with clean Ringer's solution, and tracer concentration was determined using fluorescent intensity of basal samples. Tracer diffusive transport across the epithelium [27] was determined by solving the equation at right for the monolayer permeability P to the tracer, knowing tracer concentration C on sides A and B at time t (120 min) and 0, compartment volume V, and the cell monolayer-covered co-polyester membrane area S (0.5 cm^2^) available for transport.
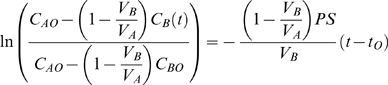



Permeability to BODIPY-tagged ouabain (radius ∼20 Å) was determined by incubating 2CLP and sham cells plated on silastic membranes for 60 minutes at a concentration of 2µM. Following the 60 minute incubation period, the apical surface was rinsed three times with DMEM, and the wells were imaged (Nikon TE-300, 10× objective) to visualize the ouabain-bound regions. This method can be used to measure paracellular permeability because the only route the BODIPY-tagged ouabain can take to access the basal membrane, where it selectively binds to Na^+^/K^+^-ATPase pumps, is through the tight junctions [26]. Previously, we have shown BODIPY-oubain flux was not altered following monolayer treatment with phenylarsine oxide (PAO, 5 µM), an inhibitor of endocytosis, indicating that motion of the tracer from apical to basal surface was paracellular in nature. In the present study to compare 2CLP and sham monolayer permeability, the stained area as a percentage of each image (N = 25 images per group: 5 isolations, 2 wells/rat, 2–3 images/well) was measured, and normalized to the percent area stained in sham monolayers. The calculated permeability to carboxyfluorescein and the area stained by BODIPY were independently compared between 2CLP and sham monolayers using a Student's t-test (p<0.5).

### Western Analysis of Signaling Activation and TJ Proteins

Cells from 2CLP and sham monolayers (N≥4 isolations for each protein) were washed with PBS and scraped from the silastic membrane in the presence of chilled radio-immunoprecipitation assay (RIPA) buffer containing 4.3 mM ethylenediaminetetraacetic acid (EDTA) and a cocktail of protease and phosphotase inhibitors, and placed on ice. Equal protein lysate was run on SDS-polyacrylamide (4–12%) gels, transferred onto polyvinylidene fluoride membranes (PVDF) and non-specific binding was blocked in TBS containing 5% non-fat powdered milk and 0.1%Tween-20 at room temperature. Membranes were probed for one of three phosphorylated MAPks (p38, ERK1/2, or JNK1/2), stripped, and then re-probed for the corresponding total proteins (all from Cell Signaling Technology, Beverly, MA). Specific activity was calculated through densitometric analysis (Kodak, Rochester, NY). Band intensities for phospho-signaling pathways were normalized by total protein levels. The normalized values from 2CLP and sham were compared using a Student's t-test (p<0.05). The same methods were used for analysis of the TJ proteins claudin 3, 4, 5, 7, 8, 18, occludin, and ZO-1 (Invitrogen, San Diego, CA). Band intensities of actin was used to normalize tight junction proteins between lanes.

### Tight Junction Protein Immunofluorescence

Cells (N = 2 wells from each of 2 isolations for each protein) were washed with phosphate-buffered saline (PBS), fixed for 15 min in 1.5% paraformaldehyde, washed again in PBS, and treated for 5 min with 0.1% Triton X-100 in PBS to permeabilize the cell membranes. Cells were blocked for 1 hour at room temperature in 5% normal goat serum (NGS), then incubated overnight with either 20 µg/ml anti-occludin, 1.0 µg/ml anti-ZO-1, or 10 µg/ml anti-claudin 4 or 18 (all antibodies from Invitrogen, San Diego, CA) in 5% NGS in PBS. After washing in PBS, the cells were incubated for 2 hour in secondary antibody (Jackson Laboratories, West Grove, PA), mounted, and imaged (Nikon TE-300).

### Inhibition of MAPk Signaling Pathways

Cell monolayers on both silastic membranes and transwell supports were serum deprived for 2 hours in DMEM with HEPES salts. Apical fluid was replaced with either the JNK inhibitor SP600125 (20 µM) or the ERK inhibitor U0126 (10 µM) (Sigma, Saint Louis, MO). Cells on silastic membranes were incubated with the inhibitor for 60 minutes, and then lysed for analysis of signaling activation and TJ protein concentration as described above (N = 9 isolations per group, 1–2 wells per isolation). Transepithelial resistance of the cells on transwell supports was measured 0, 1, 1.5, and 2 hours following the application of the inhibitor as described above. All inhibitor results were compared to DMSO controls in sham and 2CLP wells using ANOVA with post-hoc Tukey tests used for individual comparisons (p<0.05).

## Results

### Cecal Ligation and Puncture Increases Immune Activity Within 24 Hours

**Figure 1:**
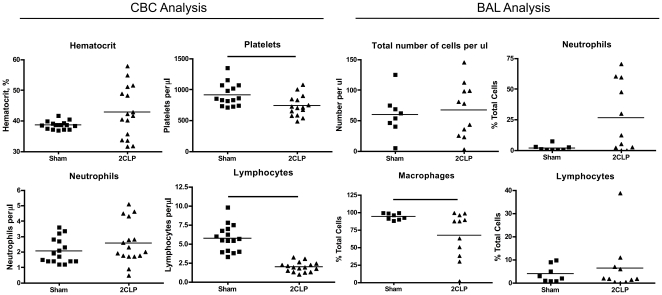
Analysis of Complete Blood Count and Bronchoalveolar lavage fluid. (Left) Complete Blood Count (CBC) data (normalized per vol or %). Elevated hematocrit (HCT) is likely related to dehydration, while decreased levels of platelets (PLT) and lymphocytes are observed with sepsis. (Right) Bronchoalveolar lavage (BAL) fluid data (expressed as % total cells). The total number of cells in the BAL was not different between sham and 2CLP, however more neutrophils and less macrophages were observed in 2CLP lungs than sham lungs. Significance (-) is defined as p<0.05 as determined by a Mann-Whitney nonparametric test.

Blood samples obtained at sacrifice were analyzed for cellular content (number of cells per unit volume). We observed that 2CLP rats had many symptoms recognized in septic patients [28], specifically platelet (737±169 and 915±185) and lymphocyte (1.96±0.64 and 5.71±1.69) were significantly lower in 2CLP animals compared to sham (mean±standard deviation, Figure 1, left). Furthermore, 2CLP animals were significantly more lethargic, with increased porphyrin staining of the eyes and nose, and internal tissues appeared dehydrated and inflamed compared to sham animals.

**Figure 2:**
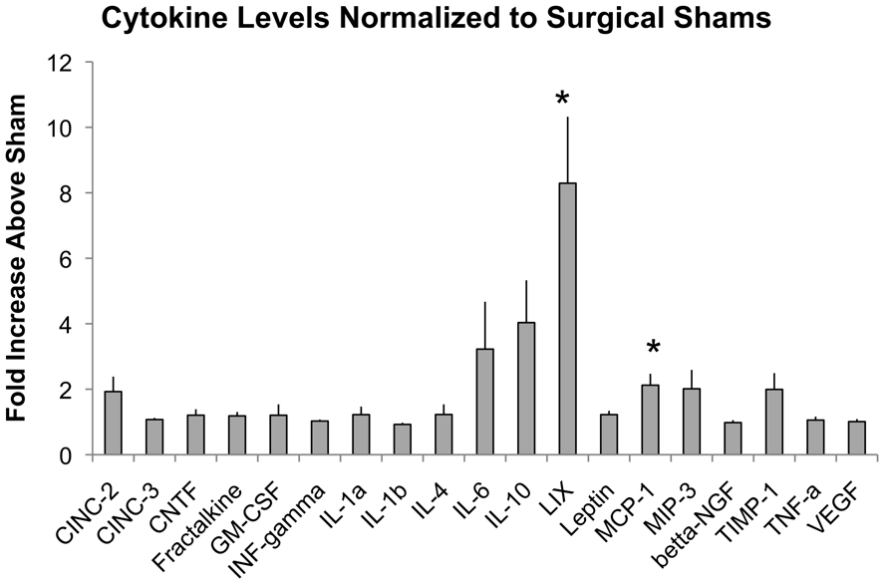
Serum cytokine levels in septic (2CLP) rats normalized to sham controls (value of 1) as determined by microarray. Increased concentrations of Cinc-2, IL-6, IL-10, LIX, MCP-1, MIP-3, and TIMP-1 show activation of the immune response to the bacterial infection. (N = 6, μ±SE).

Analysis of cytokine and chemokine expression in the blood plasma revealed significantly elevated levels of LIX and MCP-1in 2CLP compared to sham (Figure 2). Furthermore, many of the other probed cytokines including IL-6 and IL-10 were elevated in 2CLP compared to sham, however these differences did not reach statistical significance. Elevation of IL-6 has been observed in human adult and neonatal sepsis, and shown to correlate with increased mortality [29], [30]. Therefore, we conclude that the increased level of IL-6 in our model should be evaluated further in the future.

Cellular content of the BAL was also analyzed (Figure 1, right). The concentration of cells (total cells per µl) was not different between 2CLP and sham groups. The percentage of cells which were neutrophils (26.3±28.1 and 1.88 and 2.49 from 2CLP and sham respectively) increased in 2CLP animals, although this increase was not statistically significant. Furthermore, the percentage of cells which were macrophages (67.3±33.2 and 94.2±4.57) was significantly lower in 2CLP animals. Neutrophil sequestration in the lung is recognized as a symptom of sepsis, and is thought to promote organ dysfunction [31]. Therefore, we conclude our 2CLP data demonstrate both systemic (blood) and pulmonary markers consistent with sepsis.

### Alveolar Epithelial Type II Cells from 2CLP and Sham Animals Assume Type I Phenotype in Culture

**Figure 3:**
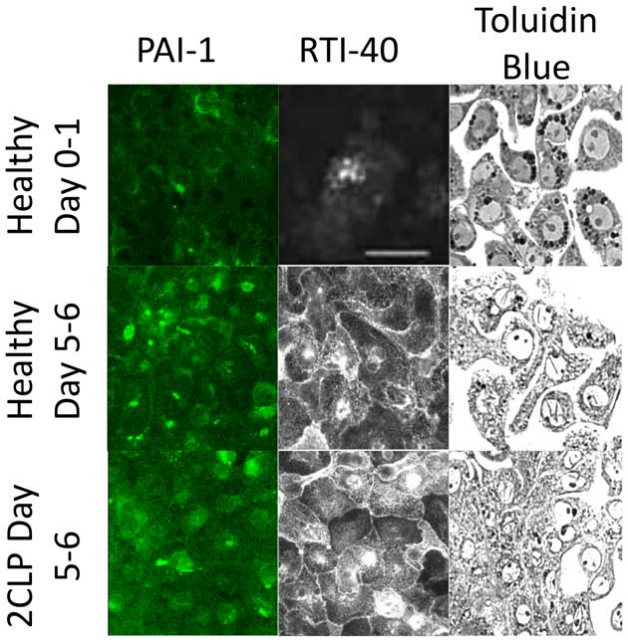
Staining for phenotypic markers of alveolar type II (Toluidin Blue stain of lamellar bodies) and type I (PAI-1, RT1-40) in freshly isolated healthy cells, healthy day 5-6 cells, and 2CLP day 5–6 cells. These images demonstrate a loss of type II markers in healthy cells and expression of type I markers by day 5. Staining in 2CLP cells on day 5–6 is similar to that in healthy cells, indicating that they have also take on a alveolar type I-like epithelial phenotype. Scale bar 50 µm.

As demonstrated previously by ourselves and others, freshly isolated alveolar type II cells cultured for a period of 5 days take on an alveolar epithelial type I-like phenotype and form confluent monolayers [26], [32], [33]. To examine if cells isolated from septic animals behave similarly, we fixed cells isolated from 2CLP animals following 5–6 days in culture and stained them with antibodies for type I phenotypic markers (PAI-1, RTI-40) and type II phenotypic marker (lamellar body marker Toluidin Blue) (Figure 3) [15], [34]. In healthy cells, we observed increased staining of all type I markers and concurrent decreased staining of lamellar bodies (Toluidin Blue) with increasing days in culture. Furthermore, similar staining patterns were observed in 2CLP monolayers following 5–6 days in culture, as PAI-1, and RTI-40 staining increased, and toluidin blue staining was diminished compared to healthy cells at 0–2 days in culture. These data show that like healthy cells, alveolar epithelial type II cells isolated from septic rats down-regulate type II phenotypic markers and up-regulate type I phenotypic markers throughout days in culture. Therefore, they can be used to assess barrier function of type I-like epithelial cells following a septic exposure.

**Figure 4:**
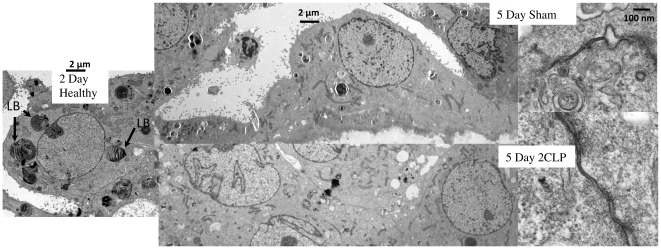
Electron micrographs (2,500×–150,000×) of a healthy cell on day 2 demonstrating the presence of lamellar (arrows) bodies in isolated alveolar type II cells, and sham and 2CLP cells on day 5 demonstrating a loss of lamellar bodies and formation of tight junctions.

Electron microscopy was utilized to investigate the presence of lamellar bodies and tight junction complexes in healthy cells following 2 days in culture and sham and 2CLP cells following 5 days in culture (Figure 4). The healthy cell on day 2 shows numerous lamellar bodies, a marker of a type II phenotype. Both 2CLP and sham cells on day 5 lack lamellar bodies. At higher magnification, micrographs of 2CLP and sham cells reveal tight junctional complexes.

### 2CLP and Sham Attachment and Spreading

**Figure 5:**
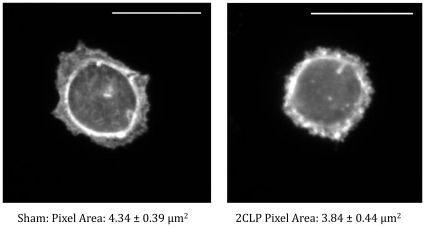
Area of sham and 2CP cells plated on fibronectin coated glass slides was analyzed on day 2. Images (60× objective) were taken of phalloidin stained actin to visualize the cell boundary. No differences in epithelial size was observed, indicated that the growth rates of sham and 2CLP cells was not significantly different. (Scale bar = 10 µm, N≥18 cells, μ ± SE).

To determine if cells isolated from 2CLP and sham animals spread at similar rates, we measured the area of cells plated at subconfluent levels on fibronectin coated glass slides. Following the 2-day culture period, the area of (phalloidin stained) 2CLP (N = 18) and sham (N = 25) cells was measured (Figure 5). After 2 days in culture we found no significant difference in the area of these cells (384±44.2 µm^2^ in the 2CLP and 433±38.7 µm^2^ in the sham), indicating that both 2CLP and sham cells adhere and spread after seeding at similar rates. By 5 days in culture on a flexible substrate, cell size is significantly increased in both 2CLP (N = 73) and sham (N = 76), with no difference between groups (667±24.4 µm^2^ in the 2CLP and 651±23.8 µm^2^ in the sham). Because cell size data were indistinguishable between 2CLP and sham at 2 and 5 days, albeit on different substrates, we conclude they spread at similar rates. Finally, we can conclude that similar numbers of cells are present in the monolayers at day 5, based on the observation that the cells are of similar size in the confluent monolayers in the 2CLP and sham populations.

### Epithelial Monolayers Composed of Cells from Septic Rats are More Permeable to Ions but not Molecular Tracers than Cells from Shams

**Figure 6:**
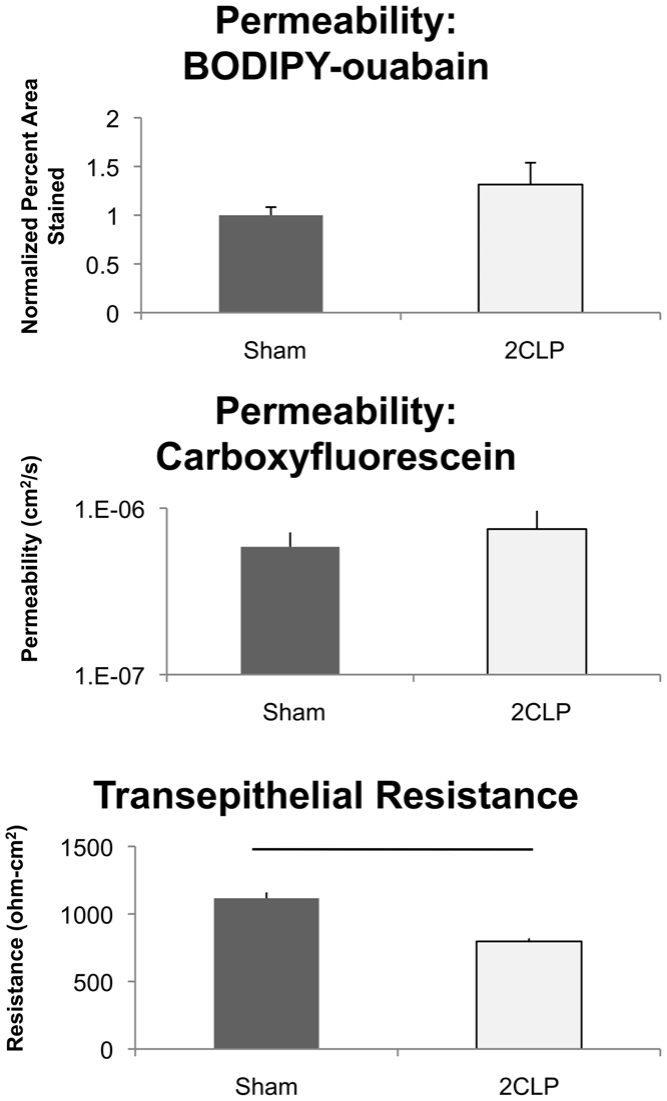
Permeability analysis of monolayers from sham and 2CLP animals. (Top) Monolayer permeability to the molecular tracer BODIPY-ouabain (∼20 Å) is shown normalized to the percent area stained in the sham monolayers. No significant differences were observed. (Middle) Monolayer permeability to the small molecular tracer Carboxyfluorescein (∼5 Å) was not significantly different between the two groups. (Bottom) Transepithelial resistance measurements show significant (p<0.05) differences between groups, and indicate that the 2CLP monolayers are more permeable to ions. (mean ± SE).

Subsequent to the 5-day culture period, we evaluated the barrier properties of the epithelial monolayer formed by 2CLP and sham cells. Monolayer permeability (BODIPY-ouabain, ∼20Å) was determined by measuring the area of the monolayer labeled with BODIPY-tagged oubain (Figure 6, top). No significant differences were observed in the normalized area (1.315±0.223 and 1.000±0.083 in 2CLP and sham, respectively), and we concluded that there were no differences in permeability to this moderate-sized tracer.

In another set of experiments the paracellular permeability of a smaller tracer (carboxyfluorescein, 5 Å), was determined (Figure 6, middle). As with the larger BODIPY-tagged ouabain tracer, we found no significant differences in permeability to carboxyfluorescein between 2CLP and sham monolayers (7.48×10^−7^±2.17×10^−7^ and 5.85×10^−7^±1.299×10^−7^ cm^2^/s in 2CLP and sham. respectively).

Furthermore, we observed a significant decrease in transepithelial resistance (TER) (769±97.1 and 1234±308 ohm-cm^2^ in 2CLP and sham, respectively) in the 2CLP monolayers compared to the sham (Figure 6, bottom). These data show the 2CLP monolayers are less resistant to ion flux than those in sham monolayers.

**Figure 7:**
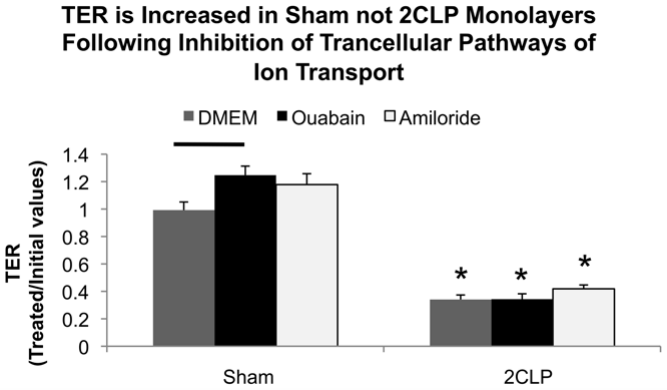
Inhibition of transcellular ion pathways modulates TER in sham not 2CLP monolayers. Following initial TER measurements, the apical fluid was replaced with 1mM amiloride (N = 9) or the basal fluid replaced with 1mM ouabain (N = 10) to block Na^+^K^+^-ATPase and ENaC activity. DMEM was used as a control media (N = 8). Ouabain, not amiloride, treatment of sham wells resulted in significant increases above DMEM controls (-). Neither treatment had a significant affect on TER in 2CLP wells. Significance defined as p<0.05.

To determine if the differences between 2CLP and sham TER was due to paracellular or transcellular pathways, we treated 2CLP and sham monolayers (N≥8 wells per treatment) with amiloride or ouabain (1mM) to block either ENaC or Na^+^K^+^-ATPase, transcellular pathways for ion motion (Figure 7). Treatment of sham monolayers with ouabain, not amiloride, significantly improved TER in sham monolayers, while neither treatment altered TER in 2CLP monolayers. From these data, and that from previous work which demonstrates little to no cell death in 2CLP monolayers following 2 days in culture, allow us to conclude that reduced TER in 2CLP monolayers are due to paracellular pathways of ion motion, possibly due to modifications in cell-cell junctions, not increased cell death [23].

### Confluent Monolayers of Epithelial Cells From Septic Animals Express Different Levels of Tight Junction Proteins Compared to Sham Monolayers

**Figure 8:**
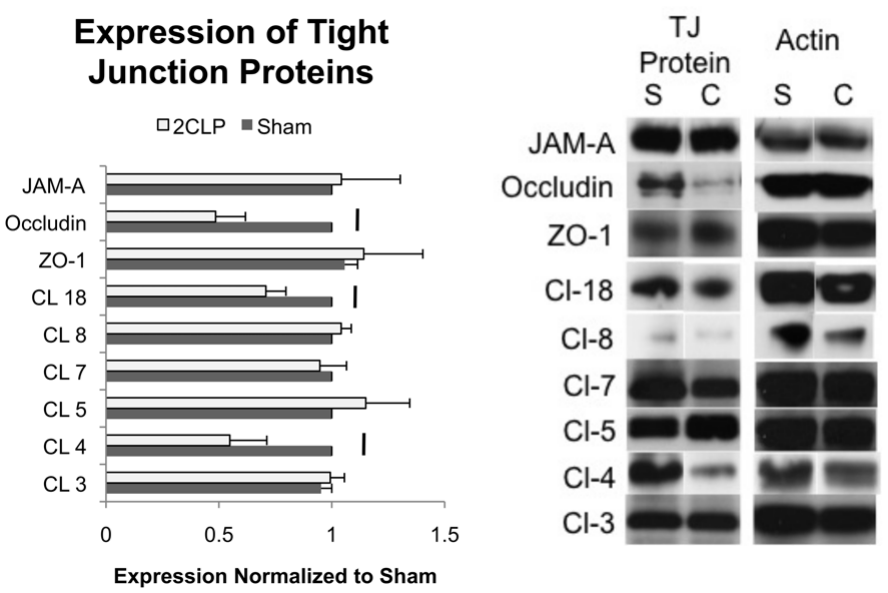
Expression levels of the TJ proteins claudin 3, 4, 5, 7, 8, 18, ZO-1, Occludin, and JAM-A were determined via western blot in sham (S) and 2CLP (C) monolayers. Significant reductions in claudin 4, 18, and occludin were observed (p<0.05, N≥3). Representative Western blots of all proteins analyzed are show along with their respective actin bands to which they were normalized. (mean ± SE).

Following 5 days in culture, monolayers of 2CLP and sham cells were lysed and tight junction protein expression was analyzed. We examined both transmembrane proteins (claudin 3, 4, 5, 7, 8, 18, occludin, and JAM-A) as well as a cytoplasmic scaffolding protein (ZO-1) (Figure 8). Claudin 4, claudin 18, and occludin were all significantly reduced in 2CLP monolayers compared to sham, while no significant differences in the expression of claudin 3, 5, 7, 8, ZO-1, and JAM-A were observed. Others have shown that reductions in claudin 4 and occludin expression can lead to increases in permeability, suggesting that the reductions in the tight junction proteins observed in our studies may be responsible for the differences in barrier function between 2CLP and sham monolayers [35], [36], [37].

### Activation of MAPk Signaling is Elevated in 2CLP Monolayers Compared to Sham

**Figure 9:**
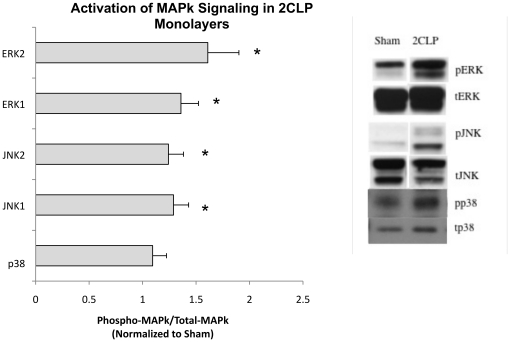
Western analysis of MAPk signaling in 2CLP and sham monolayers. We found that activation (ratio of phospho-MAPk to total MAPk) of the JNK and ERK kinases were significantly elevated in the 2CLP monolayers compared to sham (*, p<0.05, N≥12). We include representative western blots showing the phosphorylated bands as well as their respective totals. (mean ± SE).

Based on reports in the literature of MAPk signaling pathways being elevated in septic animals, we probed for phosphorylated and total JNK, p38, and ERK in cell lysates obtained from 2CLP and sham monolayers cultured for 5 days [14], [38]. We observed significant increases in the phosphorylation of JNK and ERK (normalized to total protein) in 2CLP monolayers compared to sham (Figure 9). These MAPk phosphorylation elevations are similar to those measured in freshly isolated, homogenized whole lungs after 2CLP procedures in rats [14]. Thus we conclude that alveolar epithelial cells isolated after 2CLP procedure and maintained in culture for 5 days mimic freshly isolated tissue MAPk responses.

### Inhibition of ERK, not JNK, Signaling Improves Barrier Function in 2CLP Monolayers

**Figure 10:**
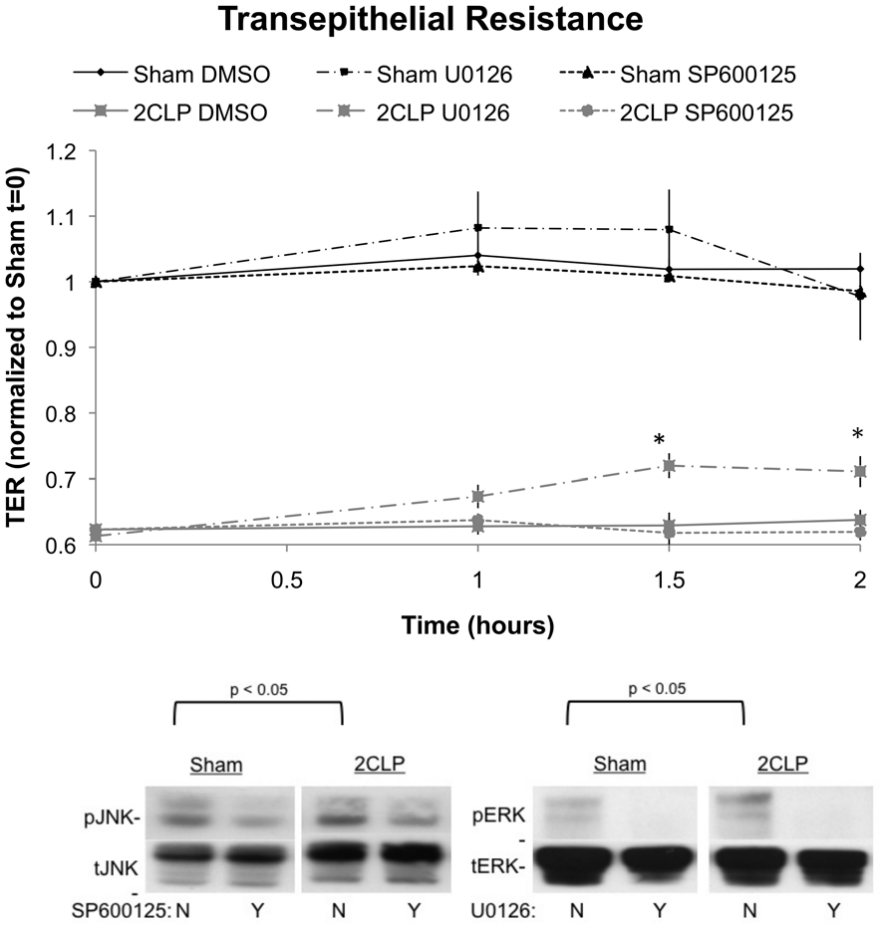
Transepithelial resistance of sham and 2CLP monolayers following treatment with either U0126 or SP600125 to inhibit ERK or JNK activation respectively. Monolayers were serum deprived for 2 hours prior to the application of the inhibitor (time = 0). Measurements were taken at 1, 1.5, and 2 hours following treatment. 2CLP monolayers have significantly lower TER values than sham, and sham monolayer TER was unaffected by any of the treatments. Only U0126 significantly improved TER in 2CLP monolayers above initial values by 1.5 hours, and this persisted to 2 hours (*, p<0.05). (μ ± SE) Western blots of lysate obtained following 2 hours of treatment demonstrating inhibition of JNK and ERK are also shown.

Specific inhibitors for ERK (U0126) and JNK (SP600125) signaling pathways were administered to 2CLP and sham monolayers, and TER was monitored over a 2 hour incubation period (Figure 10). Immediately prior to treatment (t = 0) baseline values of 2CLP monolayers were significantly lower than sham (compare to Figure 6, top). Treatment of sham monolayers with ERK or JNK inhibitors, or DMSO control, did not significantly alter TER compared with the initial value, although values did rise slightly in wells treated with the ERK inhibitor. Treatment of 2CLP monolayers with a JNK inhibitor or DMSO control did not significantly alter TER values compared to the initial level. However, treatment of 2CLP monolayers with an ERK inhibitor increased TER by 1 hour of treatment, and this increase was statistically significant compared to initial values at 1.5 hours and 2 hours of treatment. Nevertheless, TER in 2CLP monolayers did not reach sham levels with ERK inhibition at any time point, indicating that ERK inhibition was not sufficient to rescue reduced permeability with sepsis back to sham levels. Finally, these data show the increased activation of ERK, not JNK, signaling plays a role in barrier dysfunction in 2CLP monolayers.

### Inhibition of ERK Signaling Leads to a Recovery of the Tight Junction Proteins Claudin 4 and Claudin 18 in 2CLP Monolayers

**Figure 11:**
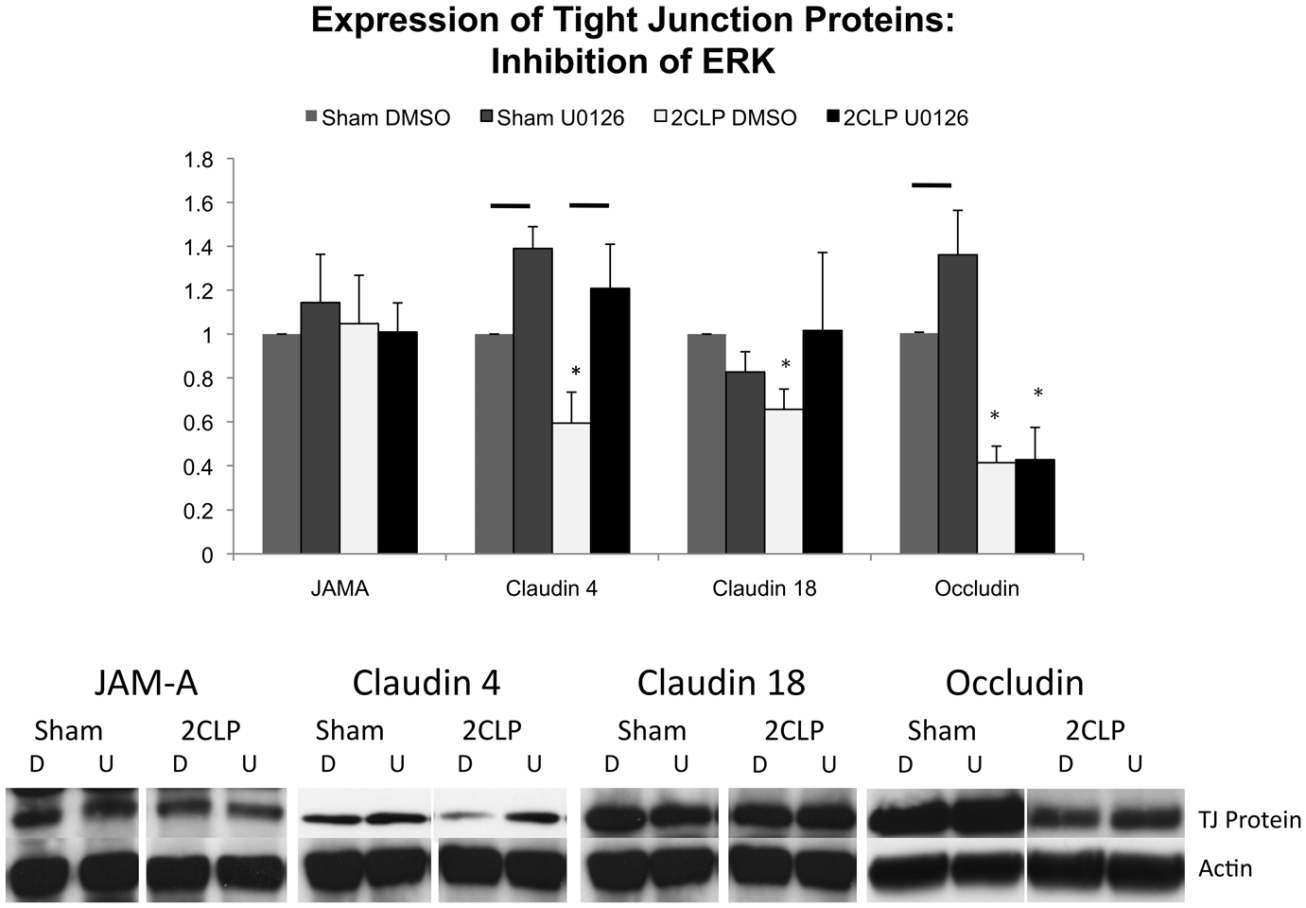
Expression of tight junction proteins are altered following 1 hour incubation with the ERK inhibitor U0126. Significant decreases in claudin 4, 18, and occludin are observed in DMSO control treated wells (*, p<0.05). U0126 leads to significant increases in claudin 4 in both sham and 2CLP monolayers, and occludin in the sham monolayers only (-, p<0.05). Claudin 18 was not significantly altered following treatment, however in 2CLP monolayers trended to be higher than DMSO controls, and was no longer significantly lower than sham levels. Also shown are representative Western blots from each group (D = DMSO, U = U0126). (μ ± SE).

We hypothesized that the improvement observed in 2CLP monolayer permeability with ERK inhibition was correlated to recovery of the deficient tight junction proteins claudin 4, claudin 18 and occludin. We lysed 2CLP and sham monolayers following 1 hour of treatment with U0126 or DMSO control, the time point at which we observe initial increases in 2CLP TER. We probed these lysates for JAM-A, claudin 4, claudin 18, and occludin (Figure 11). As in Figure 8, we did not observe differences in JAM-A expression between 2CLP and sham monolayers, while claudin 4, claudin 18, and occludin were all observed to be lower in 2CLP monolayers compared to sham (*, Figure 11). Following 1 hour treatment with U0126, JAM-A expression levels were unchanged in 2CLP and sham monolayers compared to control levels. Claudin 4 protein expression significantly increased in both 2CLP and sham monolayers compared to DMSO control. Expression of claudin 4 protein in U0126 treated 2CLP monolayers was not significantly different than sham, DMSO and U0126 treated. Claudin 18 expression increased, although not significantly, in U0126 treated, compared to DMSO treated, 2CLP monolayers, and was no longer significantly lower than sham. Treatment with U0126 significantly increased occludin expression in sham monolayers only. Because TER did not fully recover to sham levels with U0126 treatment, despite restoring claudin 4 protein expression, we conclude that claudin 4 is not solely responsible for modulating permeability.

### ERK Inhibition Alters Tight Junction Protein Localization

**Figure 12:**
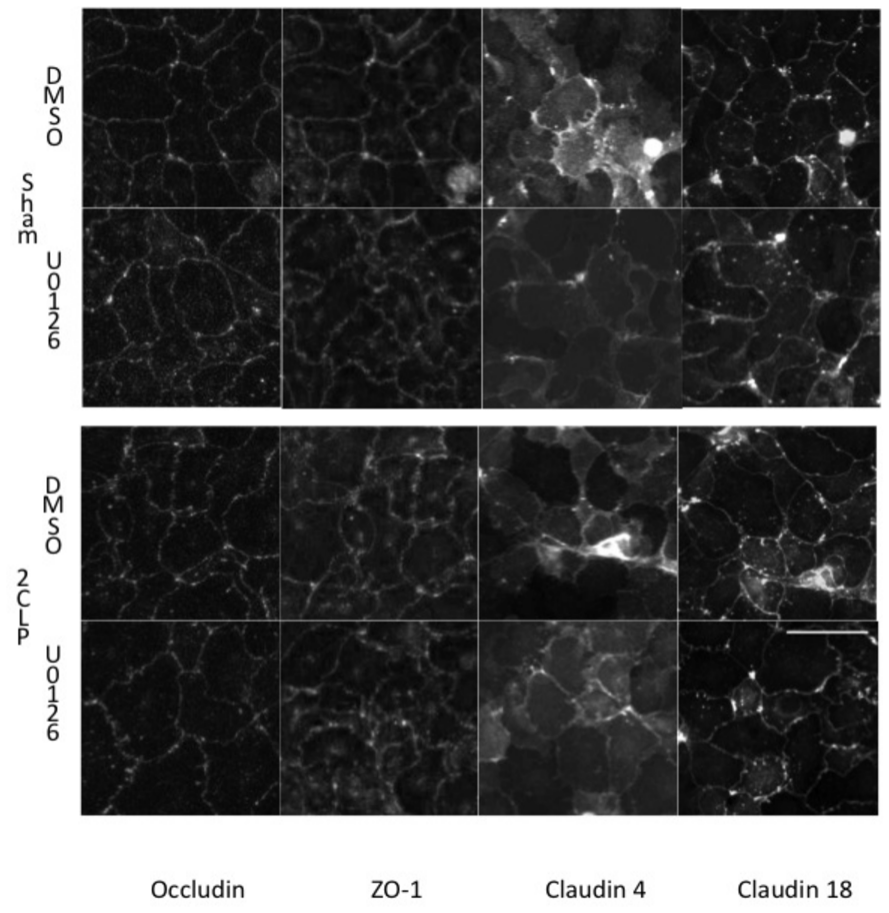
Immunofluorescent staining of the tight junction proteins occluding, ZO-1, claudin 4, and claudin 18 in 2CLP and sham monolayers treated for 1 hour with the ERK inhibitor U0126 or DMSO control. Scale bar 50 µm.

Following 1 hour of treatment with either the ERK inhibitor U0126 or DMSO control, 2CLP and sham monolayers were fixed and stained with antibodies against occludin, ZO-1, claudin 4, and claudin 18 (Figure 12). In DMSO treated wells, occludin staining was more jagged at cell-cell junctions in 2CLP compared to sham cells. Expression of claudin 4 was observed in some but not all 2CLP cells in the same monolayer, while sham cells consistently expressed the protein. Claudin 18 and ZO-1 staining pattern was similar between the two groups of cells.

Treatment with U0126 resulted in smoother junctional staining of occluding and claudin 4 in 2CLP monolayers, although some occludin junctional blebbing was observed. Intracellular staining of claudin 4 in 2CLP monolayers became more consistent with treatment. Occludin distribution in sham was not affected by treatment. No changes in claudin 18 or ZO-1 were observed in sham or 2CLP following treatment with U0126.

### Additional Mechanistic Studies

Previously, Lee *et al.* demonstrated that treatment of alveolar epithelial type II cell monolayers with edema fluid from ALI patients altered the expression of transcellular ion channels, impaired fluid clearance, and increased protein flux without altering the staining pattern of the tight junction protein ZO-1 [39]. We hypothesized that differences in MAPk activation, tight junction protein expression, and TER in 2CLP compared to sham was due to signaling molecules secreted by the 2CLP cells in culture. We obtained media incubated on 2CLP or sham monolayers for 24 hours, centrifuged it for 5 minutes at 500g to remove cellular debris, and exposed healthy cells to this conditioned media (N = 6 transwells per treatment, N = 2 isolations for ERK activation lysate). No differences were observed in either TER (2614±187.1 and 2625±326.5 ohms-cm^2^ for treated with sham and 2CLP media respectively, mean ± SE). Similarly ERK activation in healthy cells was not significantly different between the two conditioned media groups (1.29±0.62 fold increase in wells treated with 2CLP compared to those treated with sham, mean ± SE). We conclude that signaling molecules secreted by the cells in culture do not promote or contribute to the sustained septic responses observed after 5 days in culture.

In separate studies, we tested the hypothesis that increases in epidermal growth factor (EGF), a known activator of ERK signaling, might be responsible for the observed differences between 2CLP and sham populations [40]. We inhibited EGF activation in 2CLP and sham monolayers (N = 8 transwells per group) by treating them with the EGF inhibitor Tyrphoston AG-1478 (100 nM) (Cell Signaling, Danvers, MA) on days 2, 3, and 4 in culture and measured TER in sham and 2CLP wells on day 5. Values were compared to wells treated with untreated media. Interestingly, TER dropped significantly (p<0.05) in the sham monolayers (1003±72.3 and 578.3±41.7 ohms-cm^2^ in untreated and treated wells respectively, mean ± SE) with treatment. However, in 2CLP monolayers, TER (263.8±24.8 and 330.3±70.1 ohms-cm^2^ in untreated and treated wells respectively, mean ± SE) was unaffected. We conclude that EGF activation could be important for formation and maintenance of “tight” barrier function in healthy cells. The literature supports the hypothesis of EGF regulation of tight junction function, however there is ambiguity as to weather EGF activation improves or impairs barrier function [41], [42]. Regardless, EGF inhibition did not improve TER in cells obtained from septic animals.

## Discussion

Current animal models for sepsis utilize instillation of bacterial pathogens, or their components (e.g. LPS, an outer membrane component of gram-negative bacteria) into either the blood stream or peritoneum to produce a systemic up-regulation of inflammatory signaling *in vivo* [43], [44], [45]. A limitation of animal models is they cannot be used to elucidate the mechanisms by which sepsis can modulate a single cell type, such as the alveolar epithelium. For this reason, culture models of sepsis have been developed which utilize incubation of healthy cells with bacteria, endotoxin (LPS), or exogenous inflammatory mediators such as TNFα or IL-6 [19], [21], [46], [47]. Sepsis *in vivo*, however, involves systemic expression of multiple inflammatory signaling pathways, which cannot be replicated in culture [28]. The *in vitro* model presented in this communication combines the advantages of a well characterized animal model (initial systemic event simulating numerous inflammatory and anti-inflammatory signaling cascades) with those of a culture model (high specificity, high throughput), resulting in a preparation that can be used to study the mechanisms by which sepsis modulates alveolar epithelial function [45], [48].

We show that after 5 days in culture, alveolar epithelial cells isolated from 2CLP animals retain features of reported to be found *in vivo* following 2CLP, such as neutrophil accumulation in the lung, increased MAPk activation and epithelial permeability [14], [49], [50], [51]. The observation of cellular memory, or retention of environmental properties following isolation, has been reported previously. Fernandez *et. al.* showed that alveolar epithelial cells isolated from alcohol-fed rats, subsequently cultured for 7 days without alcohol retained tight junction features present in freshly isolated whole lung lysates, including decreased expression of claudin 1 and claudin 7, and increased expression of claudin 5 [52].

Following isolation and culture of the septic cells, we showed that while phenotype (at day 5) and cell size (at day 2 and 5) of these cells were similar to that of sham or healthy cells, expression of tight junction proteins claudin 4, claudin 18, and occludin were lower and TER was reduced in the septic monolayer. Furthermore, phosphorylation of MAPk signaling pathways was significantly increased in cells isolated from septic animals after 5 days in culture. These observations are supported by published observations from other *in vivo* models of sepsis [53], [54]. Specifically, Shen *et. al.* demonstrated MAPk activation (ERK and JNK) in the lungs of mice 6 hours following 2CLP, and show data implicating these pathways in the expression of inflammatory mediators such as IL-6, IL-1beta, and TNF-alpha and reductions in blood gas levels indicating loss of proper gas exchange across the epithelium [14]. In addition, *in vivo* alterations of the tight junction complex have been previously reported in the epithelial monolayer of mice intestine following 2CLP [55]. Our *in vitro* model recreates these *in vivo* alterations of lung epithelial cells following 2CLP, demonstrating its potential as an *in vitro* preparation appropriate for the study of sepsis.

We find that in 2CLP monolayers cultured from septic animals, inhibiting ERK signaling can partially rescue the increased permeability of septic monolayers. Kevil *et. al.* showed ERK regulation of H_2_O_2_ induced occludin redistribution and permeability increases in endothelial cells [56]. Similarly, we find that ERK inhibition reversed the loss of tight junction proteins claudin-4 and claudin-18 in 2CLP monolayers and partially rescued TER. These results are supported by previous evidence that claudin-4 is a restrictive tight junction protein to ion flux across MDKC monolayers and that reductions in claudin 18 correlate with increased protein flux across alveolar epithelial type II monolayers [57], [58]. Interestingly, ERK inhibition did not affect occludin concentration, in either septic or control monolayers. The differences between our study and that of Kevil *et. al.* could be due to the different injury model or different cell type utilized in the individual studies.

While this novel model of a septic alveolar epithelium provides a convenient experimental platform, it is not without limitations. Most importantly, our model is composed of only alveolar type I-like cells, and lacks alveolar type II cells. The type II cell plays an integral role in epithelial function, as it is a surfactant producing cell which has been shown to interact with neighboring type I-like cells [59]. Furthermore, the fibronectin coated elastic substrate on which our cells were seeded does not exactly model the complex basement membrane of the alveolar interstitium. A recent study has shown a significant effect of the substrate coating on epithelial function, which should be considered when interpreting data obtained using this model [60]. Finally, as with any *in vitro* model, all findings might not translate back to the *in vivo* setting, and therefore will require validation in an animal model.

In summary, we have developed a novel primary cell culture model of a septic alveolar epithelium that incorporates the complexity of septic injury *in vivo* with the specificity of an *in vitro* monoculture study. We demonstrate that our model reproduces *in vivo* observations of signaling activation and tight junction protein alterations, and show the ability to modify monolayer barrier function through inhibition of these pathways *in vitro*. This model provides a unique preparation for probing the mechanisms by which sepsis alters the alveolar epithelium.

## References

[1] Arroliga AC, Ghamra ZW, Perez Trepichio A, Perez Trepichio P, Komara JJ (2002). Incidence of ARDS in an adult population of northeast Ohio.. Chest.

[2] Thomsen GE, Morris AH (1995). Incidence of the adult respiratory distress syndrome in the state of Utah.. Am J Respir Crit Care Med.

[3] McIntyre RC, Pulido EJ, Bensard DD, Shames BD, Abraham E (2000). Thirty years of clinical trials in acute respiratory distress syndrome.. Crit Care Med.

[4] Milberg JA, Davis DR, Steinberg KP, Hudson LD (1995). Improved survival of patients with acute respiratory distress syndrome (ARDS): 1983–1993.. JAMA.

[5] Wheeler AP, Bernard GR (2007). Acute lung injury and the acute respiratory distress syndrome: a clinical review.. Lancet.

[6] Rubenfeld GD, Caldwell E, Peabody E, Weaver J, Martin DP (2005). Incidence and outcomes of acute lung injury.. N Engl J Med.

[7] Fein AM, Calalang-Colucci MG (2000). Acute lung injury and acute respiratory distress syndrome in sepsis and septic shock.. Crit Care Clin.

[8] Eisner MD, Thompson T, Hudson LD, Luce JM, Hayden D (2001). Efficacy of low tidal volume ventilation in patients with different clinical risk factors for acute lung injury and the acute respiratory distress syndrome.. Am J Respir Crit Care Med.

[9] Altschule MD (1986). Pulmonary edema induced by sepsis.. Chest.

[10] Lindert J, Perlman CE, Parthasarathi K, Bhattacharya J (2007). Chloride-dependent secretion of alveolar wall liquid determined by optical-sectioning microscopy.. Am J Respir Cell Mol Biol.

[11] Perlman CE, Bhattacharya J (2007). Alveolar expansion imaged by optical sectioning microscopy.. J Appl Physiol.

[12] Uhlig U, Haitsma JJ, Goldmann T, Poelma DL, Lachmann B (2002). Ventilation-induced activation of the mitogen-activated protein kinase pathway.. Eur Respir J.

[13] Mazzon E, Cuzzocrea S (2007). Role of TNF-alpha in lung tight junction alteration in mouse model of acute lung inflammation.. Respir Res.

[14] Shen L, Mo H, Cai L, Kong T, Zheng W (2009). Losartan prevents sepsis-induced acute lung injury and decreases activation of nuclear factor kappaB and mitogen-activated protein kinases.. Shock.

[15] McElroy MC, Kasper M (2004). The use of alveolar epithelial type I cell-selective markers to investigate lung injury and repair.. Eur Respir J.

[16] Zhou G, Dada LA, Wu M, Kelly A, Trejo H (2009). Hypoxia-Induced Alveolar Epithelial-Mesenchymal Transition Requires Mitochondrial Ros and Hypoxia-Inducible Factor 1.. Am J Physiol Lung Cell Mol Physiol.

[17] Cavanaugh KJ, Cohen TS, Margulies SS (2006). Stretch increases alveolar epithelial permeability to uncharged micromolecules.. Am J Physiol Cell Physiol.

[18] Cohen TS, Cavanaugh KJ, Margulies SS (2008). Frequency and peak stretch magnitude affect alveolar epithelial permeability.. Eur Respir J.

[19] Escobar GA, McIntyre RC, Moore EE, Gamboni-Robertson F, Banerjee A (2006). Clathrin heavy chain is required for TNF-induced inflammatory signaling.. Surgery.

[20] Li Q, Zhang Q, Wang M, Zhao S, Ma J (2008). Interferon-gamma and tumor necrosis factor-alpha disrupt epithelial barrier function by altering lipid composition in membrane microdomains of tight junction.. Clin Immunol.

[21] Baines DL, Albert AP, Hazell MJ, Gambling L, Woollhead AM (2009). Lipopolysaccharide modifies amiloride-sensitive Na(+) transport processes across human airway cells: role of mitogen-activated protein kinases ERK 1/2 and 5.. Pflugers Arch.

[22] Soong G, Parker D, Magargee M, Prince AS (2008). The type III toxins of Pseudomonas aeruginosa disrupt epithelial barrier function.. J Bacteriol.

[23] Levine GK, Deutschman CS, Helfaer MA, Margulies SS (2006). Sepsis-induced lung injury in rats increases alveolar epithelial vulnerability to stretch.. Crit Care Med.

[24] Tschumperlin DJ, Margulies SS (1998). Equibiaxial deformation-induced injury of alveolar epithelial cells in vitro.. Am J Physiol.

[25] Tice LW, Carter RL, Cahill MC (1977). Tracer and freeze fracture observations on developing tight junctions in fetal rat thyroid.. Tissue Cell.

[26] Cavanaugh KJ, Margulies SS (2002). Measurement of stretch-induced loss of alveolar epithelial barrier integrity with a novel in vitro method.. Am J Physiol Cell Physiol.

[27] Kim KJ, Crandall ED (1982). Effects of lung inflation on alveolar epithelial solute and water transport properties.. Journal of Applied Physiology: Respiratory, Environmental & Exercise Physiology.

[28] Riedemann NC, Guo RF, Ward PA (2003). The enigma of sepsis.. J Clin Invest.

[29] Lam HS, Ng PC (2008). Biochemical markers of neonatal sepsis.. Pathology.

[30] Damas P, Ledoux D, Nys M, Vrindts Y, De Groote D (1992). Cytokine serum level during severe sepsis in human IL-6 as a marker of severity.. Ann Surg.

[31] Zemans RL, Colgan SP, Downey GP (2009). Transepithelial migration of neutrophils: mechanisms and implications for acute lung injury.. Am J Respir Cell Mol Biol.

[32] Borok Z, Danto SI, Zabski SM, Crandall ED (1994). Defined medium for primary culture de novo of adult rat alveolar epithelial cells.. In Vitro Cell Dev Biol Anim.

[33] Dobbs LG, Williams MC, Gonzalez R (1988). Monoclonal antibodies specific to apical surfaces of rat alveolar type I cells bind to surfaces of cultured, but not freshly isolated, type II cells.. Biochim Biophys Acta.

[34] Fehrenbach H (2001). Alveolar epithelial type II cell: defender of the alveolus revisited.. Respir Res.

[35] Van Itallie CM, Holmes J, Bridges A, Gookin JL, Coccaro MR (2008). The density of small tight junction pores varies among cell types and is increased by expression of claudin-2.. J Cell Sci.

[36] Colegio OR, Van Itallie C, Rahner C, Anderson JM (2003). Claudin extracellular domains determine paracellular charge selectivity and resistance but not tight junction fibril architecture.. Am J Physiol Cell Physiol.

[37] Tortolani PJ, Kaufman HS, Nahabedian MY, Frassica FJ (1999). Pericapsular fistula of the hip after radiation therapy and resection of a rectal carcinoma. A case report.. J Bone Joint Surg Am.

[38] Singleton KD, Beckey VE, Wischmeyer PE (2005). GLUTAMINE PREVENTS ACTIVATION OF NF-kappaB AND STRESS KINASE PATHWAYS, ATTENUATES INFLAMMATORY CYTOKINE RELEASE, AND PREVENTS ACUTE RESPIRATORY DISTRESS SYNDROME (ARDS) FOLLOWING SEPSIS.. Shock.

[39] Lee JW, Fang X, Dolganov G, Fremont RD, Bastarache JA (2007). Acute lung injury edema fluid decreases net fluid transport across human alveolar epithelial type II cells.. J Biol Chem.

[40] Duquesnes N, Vincent F, Morel E, Lezoualc'h F, Crozatier B (2009). The EGF receptor activates ERK but not JNK Ras-dependently in basal conditions but ERK and JNK activation pathways are predominantly Ras-independent during cardiomyocyte stretch.. Int J Biochem Cell Biol.

[41] Flores-Benitez D, Rincon-Heredia R, Razgado LF, Larre I, Cereijido M (2009). Control of tight junctional sealing: roles of epidermal growth factor and prostaglandin E2.. Am J Physiol Cell Physiol.

[42] Basuroy S, Seth A, Elias B, Naren AP, Rao R (2006). MAPK interacts with occludin and mediates EGF-induced prevention of tight junction disruption by hydrogen peroxide.. Biochem J.

[43] Villar J, Ribeiro SP, Mullen JB, Kuliszewski M, Post M (1994). Induction of the heat shock response reduces mortality rate and organ damage in a sepsis-induced acute lung injury model.. Crit Care Med.

[44] Remick DG, Ward PA (2005). Evaluation of endotoxin models for the study of sepsis.. Shock.

[45] Rittirsch D, Hoesel LM, Ward PA (2007). The disconnect between animal models of sepsis and human sepsis.. J Leukoc Biol.

[46] Deng Y, Ren X, Yang L, Lin Y, Wu X (2003). A JNK-dependent pathway is required for TNFalpha-induced apoptosis.. Cell.

[47] Gomez MI, Sokol SH, Muir AB, Soong G, Bastien J (2005). Bacterial induction of TNF-alpha converting enzyme expression and IL-6 receptor alpha shedding regulates airway inflammatory signaling.. J Immunol.

[48] Weiss YG, Bouwman A, Gehan B, Schears G, Raj N (2000). Cecal ligation and double puncture impairs heat shock protein 70 (HSP-70) expression in the lungs of rats.. Shock.

[49] Matute-Bello G, Frevert CW, Martin TR (2008). Animal models of acute lung injury.. Am J Physiol Lung Cell Mol Physiol.

[50] Liaw WJ, Chen TH, Lai ZZ, Chen SJ, Chen A (2005). Effects of a membrane-permeable radical scavenger, Tempol, on intraperitoneal sepsis-induced organ injury in rats.. Shock.

[51] Hubbard WJ, Choudhry M, Schwacha MG, Kerby JD, Rue LW (2005). Cecal ligation and puncture.. Shock.

[52] Fernandez AL, Koval M, Fan X, Guidot DM (2007). Chronic alcohol ingestion alters claudin expression in the alveolar epithelium of rats.. Alcohol.

[53] Arndt PG, Young SK, Lieber JG, Fessler MB, Nick JA (2005). Inhibition of c-Jun N-terminal kinase limits lipopolysaccharide-induced pulmonary neutrophil influx.. Am J Respir Crit Care Med.

[54] Asaduzzaman M, Wang Y, Thorlacius H (2008). Critical role of p38 mitogen-activated protein kinase signaling in septic lung injury.. Crit Care Med.

[55] Li Q, Zhang Q, Wang C, Liu X, Li N (2009). Disruption of tight junctions during polymicrobial sepsis in vivo.. J Pathol.

[56] Kevil CG, Oshima T, Alexander B, Coe LL, Alexander JS (2000). H(2)O(2)-mediated permeability: role of MAPK and occludin.. Am J Physiol Cell Physiol.

[57] Fang X, Lee J-W, Neyrink A, Gonzales LW, Ballard PL (2009). Claudin-18 May Contribute to the Increase of Protein Permeability in Cultured Human Alveolar Epithelial Type II Cells Exposed to Proinflammatory Cytokines.. FASEB J.

[58] Van Itallie C, Rahner C, Anderson JM (2001). Regulated expression of claudin-4 decreases paracellular conductance through a selective decrease in sodium permeability.. J Clin Invest.

[59] Patel AS, Reigada D, Mitchell CH, Bates SR, Margulies SS (2005). Paracrine stimulation of surfactant secretion by extracellular ATP in response to mechanical deformation.. Am J Physiol Lung Cell Mol Physiol.

[60] Koval M, Ward C, Findley MK, Roser-Page S, Helms MN Extracellular matrix influences alveolar epithelial claudin expression and barrier function.. Am J Respir Cell Mol Biol.

